# Associations of time spent on different types of digital media with self-rated general and mental health in Swedish adolescents

**DOI:** 10.1038/s41598-024-83951-x

**Published:** 2025-01-06

**Authors:** Helena Frielingsdorf, Victoria Fomichov, Ingrid Rystedt, Sofia Lindstrand, Laura Korhonen, Hanna Henriksson

**Affiliations:** 1 Department of Health, Medicine and Caring Sciences, Linköping University, Linköping, Sweden; 2 Unit for Strategic Healthcare, Region Östergötland, Linköping, Sweden; 3 Barnafrid and Department of Biomedical and Clinical Sciences, Linköping University, Linköping, Sweden; 4 Center for Social and Affective Neuroscience, Department of Biomedical and Clinical Sciences, Linköping University, Linköping, Sweden; 5 Department of Child and Adolescent Psychiatry, Linköping University, Linköping, Sweden; 6 Department of Biomedical and Clinical Sciences, Linköping University, Linköping, Sweden

**Keywords:** Adolescents, Digital media, Screen time, Mental health, Self-reported health, Risk factors, Health policy, Public health, Health care, Disease prevention

## Abstract

Although previous studies have suggested an association between digital media use and health, detailed knowledge about how different types of digital media impact adolescent health is limited. This cross-sectional population-based study explored the relationship between time spent on various digital media and adolescents’ self-rated general and mental health. The study included 3566 Swedish high school students aged 16–17 years. Associations between time spent on digital media (social media, gaming, watching movies/series/video clips etc. and digital schoolwork) and self-rated health outcomes (general health, self-esteem, symptoms of worry/anxiety and low mood/depression, trust in other people, head/neck/shoulder pain and sleep quality) was assessed. Statistical analyses employed logistic regression models adjusted for covariates (sociodemographic variables and health behaviours). In the adjusted models, spending very high amounts of time (≥ 6 h/day) on any digital media, except schoolwork, was associated with poor self-esteem, symptoms of low mood/depression and poor sleep (ORs 1.35–2.93, *p* < 0.05). Spending six hours or more on digital media was also associated with worry/anxiety (for social media and gaming), head/neck/shoulder pain (for social media, watching movies/series/video clips and schoolwork), poor general health (for gaming and watching movies/series/video clips) and low trust (for gaming) (ORs 1.39–3.18, *p* < 0.05). High amounts of time (4–5 h/day) of watching movies/series/video clips was associated with daily symptoms of low mood/depression, neck/shoulder pain, and low trust in other people (ORs 1.46, 1.41, and 1.32, *p* < 0.05). This study indicates a dose-response relationship between digital media use and adverse health outcomes in adolescents, where adverse associations start to appear after 4–5 h, particularly for watching movies/series/video clips. However, further longitudinal studies, randomized controlled trials and public health interventions focused on healthy and balanced use of digital media are warranted.

**Supplementary Information:**

The online version contains supplementary material available at 10.1038/s41598-024-83951-x.

## Introduction

Depression and other mental health problems among adolescents have increased globally in recent decades^1^. In Sweden, both the number of adolescents reporting symptoms of mental distress and those diagnosed with depression and anxiety have risen markedly since the 1990s^2^. Mental health problems during adolescence can cause suffering and incomplete schooling and confer an increased risk of developing a mental disorder later in life^1,3^. Preventing and mitigating adverse mental health outcomes among adolescents is a major public health challenge.

Digital media can be defined as anything that is transmitted over the internet or computer networks, on any device intended for the purpose^4^. A rapid increase in the use of digital media has paralleled the increase in mental health problems among adolescents. This fact has raised questions about the role of digital media as a risk factor for mental health problems. Adolescence is a time of physical, cognitive, social, and emotional development^5^. Behaviours such as digital media usage in this age may have effects on lifelong health. Additionally, adolescence is a period when many young individuals increase their engagement in different digital media, further highlighting the need to understand its effects on their health and well-being.

Adolescents’ use of digital media has increased and diversified at an unprecedented rate in recent decades. Twenty years ago, smartphones did not exist; today, almost all (97–100%) Swedish adolescents have smartphones^6^. Many adolescents worldwide spend more time on digital platforms, including smartphones, tablets, and computers, than on schoolwork and being with their peers or parents^7^. In a Swedish survey from 2021, a majority of Swedish adolescents reported using social media more than 3 h per day^6^. A similar study from the USA showed that up to 45% of adolescents reported being online almost constantly^7^.

The use of digital media can impact mental well-being in many ways, in both positive and negative directions. Digital media use associated with positive health effects includes writing text messages with close friends, exposure to pictures of nature and positive body images and positive feedback on pictures^8^. Participating in social media communities and social support groups can also promote health^9^. Longitudinal associations between higher screen time and poor sleep, and to some extent, depressive symptoms, have been established in large international meta-analyses and review articles^10–12^. Social media use has been associated with poor body image in longitudinal studies of adolescents^13,14^. In cross-sectional studies, total daily screen use higher than 2–4 h, has been associated with a large number of adverse general and mental health outcomes, including overweight and obesity, backache, headache and symptoms of depression and anxiety^15–19^, and social media has been associated with symptoms of depression, anxiety^17,20^ and poor sleep^10,12^. Also, more than two hours spent on gaming per day has been associated with symptoms of depression^17,20^, and to a varying degree with overweight and obesity as well as poor sleep^10,16^. Furthermore, screen time has been linked to poorer self-rated general health (self-rated general health reflects an individual’s overall perception of their physical and mental health) which is of importance considering that self-rated health has been strongly linked to later health outcomes^21^. Trust in others has been linked to different health behaviours^22^ as well as a number of measures of poorer health in the population^23^, making it relevant to examine its relationship with digital media usage. Consequently, it is important to investigate how digital media usage is related to various general and mental health outcomes, such as self-rated general health, self-esteem, symptoms of worry and anxiety, low mood and depression, trust in others, head/neck/shoulder pain, and sleep quality.

Adverse effects of digital media can be divided into *direct effects* caused by the content or as a consequence of the screen and its functions, such as effects of multitasking, push-notices and blue light, and *indirect effects* caused by consequences of digital media “stealing” time from other activities, such as sleep, physical activity and schoolwork, i.e., so-called displacement effects^24^. To date, an increasing number of studies have pointed out the necessity of a deeper understanding of how the type and content of digital media usage might affect adolescent health^11,25,26^. In 2020, more than half of Swedish adolescents reported spending “too much time” on their smartphones and would like to spend more time on other activities^6^. Parents and adolescents are asking for more precise guidelines about screen time to avoid potentially harmful effects of digital media and want to know how much screen time is considered “too much”^6^. However, it is difficult to determine the exact cut-off between well-balanced use and more problematic use that has negative consequences. In a large meta-analysis, some studies found adverse health effects after more than half an hour of screen use, and in other studies no effects were seen until after three and a half hours of use per day^20^. Evidently, although several studies have reported an association between screen time and different mental health outcomes^27–29^, clear definitions of cut-offs for when digital media use can cause adverse effects on general or mental health are still lacking. Therefore, it is important to investigate how the amount of time spent on digital media relates to different health outcomes.

Many previous studies reporting associations between digital media and health indicators have focused on either time spent on all types of digital media combined or specifically time spent on social media or gaming^10,12,16,17,20,30–34^. Also, some categories of digital media can be more interactive, like gaming, and others are usually more passive, like watching videos and clips, and may therefore have different effects on health. Therefore, more research is needed on how various types of digital media, such as social media, gaming, watching movies/series/video clips, and digital schoolwork, might impact health differently. Furthermore, the associations between time spent on digital media and different health outcomes are complex and influenced by a multitude of factors, including cultural, socioeconomic and life-style factors. Indeed, large review articles on the topic point out that the relationship between these factors are not studied enough^12,35^. This highlights the need to further investigate the associations between digital media and health across different cultures.

Hence, the present study aims to further elucidate the relationships between how much time Swedish adolescents spend on digital media (social media, gaming, watching movies/series/video clips etc., and digital schoolwork) and self-reported general and mental health. In order to address this aim we utilized data from the population-based web survey “Om mig” (“About Me”), which contains data about time spent on different types of digital media alongside questions about general and mental health in Swedish adolescents.

## Methods

### Study design, participants and setting

In this study, we used data from the population-based web survey “Om mig” (“About Me”) conducted annually in Östergötland County, Sweden, as part of public health surveillance^36^. The “Om mig” survey is a voluntary survey that is offered to all adolescents in year two of high school. At this stage students are generally between 16 and 17 years old. The students completed the survey online during school hours. They were also allowed to complete the survey during their free time if they preferred or could not participate during the scheduled session. The survey includes questions about life and health, covering family and friends, health and lifestyle, tobacco and alcohol, school, and leisure time. In 2019, questions about time spent on digital media were added.

In the present study, we utilized data from the 2019 survey conducted between September 16th and October 25th, 2019. Forty-one high schools in Östergötland County (71% of all schools) choose to administer the survey to their students. In the participating schools, 4139 adolescents (75%) agreed to participate in the survey. Of them, 573 adolescents lacked complete data on screen time and were excluded from the analysis. Hence, a total of 3566 adolescents were included in this study. The survey is based on youth participation, where adolescents take part in creating the questions to make them understandable and relevant. This is accomplished through interviews with adolescents, individually and in focus groups. After creating the questions in the survey, they are also tested on adolescents to make sure they are feasible and understood correctly. All questions were developed for the “Om mig” survey and not specifically for this study.

All adolescents received written information about the study and were informed that participation was voluntary and that their answers might be used in research. Participants answered the survey anonymously. Informed consent to participate in the study was ascertained by the submission of a completed questionnaire. The Swedish Ethical Review Authority (ref no Dnr 2014/298 -31; Dnr 2021–04953) approved this study and its procedures for obtaining informed consent from the study subjects. This study was conducted according to the guidelines of the Declaration of Helsinki.

### Measures and variables

#### Time spent on digital media

Unfortunately, there has been very little research validating measures of self-reported screen time among adolescents^37,38^ making it difficult to use pre-defined questions in this topic. However, the “Om mig” survey contained questions about digital media that were developed based on interviews with adolescents about their experiences of screen time usage. During these interviews, it became evident that digital media use mainly consisted of usage of social media, gaming, watching movies/series/video clips, and digital schoolwork. Consequently, the survey included questions about these specific activities. The questions were pilot-tested on adolescents before usage to make sure they were feasible and understood correctly. Therefore, time spent on digital media was measured as the reported daily time spent in different digital media categories: social media, watching movies/series/video clips etc., gaming and using digital tools for schoolwork. Time spent in the different categories of digital media was measured using categorical scales where adolescents responded to the question by choosing between nine different time categories: not at all, 30 min, 1 h, 2 h, 3 h, 4 h, 5 h, 6 h and 7 h or more. After obtaining the data, time spent on different digital media was categorized into four categories: low, moderate, high and very high, corresponding to “1 h or less”, “2 to 3 h”, “4 to 5 h”, and “≥ 6 h”, respectively. As common in previous studies^39–41^, we included data from all individuals, regardless of their total digital media usage.

#### Health outcomes

Self-rated health outcomes were measured using categorical scales with four or five response categories. The following self-rated health outcomes were used: general health, self-esteem, daily symptoms of worry/anxiety and low mood/depression, trust in other people, headache, daily neck/shoulder pain and sleep quality. General health was measured using the question “How would you say your health is?” and included five response categories: very good, good, fair, poor, and very poor. This self-reported question has proven valid in adequately measuring “general health”^21,42,43^. Sleep was measured using the question “How would you say your sleep is?” and included four response categories: very good, quite good, quite poor, and very poor. In the analyses, responses about health and sleep were dichotomized into poor (quite poor, poor, and very poor) and not poor (very good, good, quite good, and fair). Daily symptoms of worry/anxiety, low mood/depression, headache, and neck/shoulder pain were measured using the question “How often have you had any of these problems during this semester…?” and included four response categories: almost daily, a few times per week, a few times per month and seldom or never. “This semester” refers to mid-August 2019 (school opening) to the date adolescents completed the survey, which varied between September 16th and October 25th. As a result, the recall period ranged from 4 to 10 weeks. In the analyses, the responses were dichotomized into daily and not daily (a few times per week, a few times per month and seldom or never). Self-esteem was measured using the question “How often do you feel that you are good enough just the way you are?” and included five response categories: always, often, sometimes, rarely, and never. In the analyses, the responses were dichotomized into poor (rarely and never) and not poor (always, often, sometimes). Trust in other people was measured using the question “How much do you trust other people in general?” and included four response categories: a lot, quite a lot, fairly little, and a little. In the analyses, they were dichotomized into low (a little, fairly little) and not low (a lot, quite a lot). The questions about symptoms of worry/anxiety and low mood/depression are similar to the questions in the Kessler-6 screening tool, which provides an accurate and valid measure of mental distress^44,45^. Also, very similar questions for symptoms of low mood/depression, self-esteem, headache and back pain are found in The Health Behaviour in School-aged Children (HBSC) study, which is a large cross-national research study about health and well-being in children involving data from 51 countries^46^.

#### Sociodemographic and health behaviour covariates

Several covariates were considered. We hypothesized that sociodemographics such as gender, high school program, origin, and family finances may confound the associations between time spent on digital media and health outcomes given their associations with health^23^, and were therefore used as covariates. Gender was defined as “male”, “female”, or “other”. Origin had two possible values: “Swedish” (born in Sweden/Nordic countries with at least one parent born in Sweden/Nordic countries) and “non-Swedish” (not born in Sweden/Nordic countries or two parents born abroad). Family finances were measured using the question “How often do you experience that your family can afford to do/buy the same things as most other students at your school?” and included five response categories: always, often, sometimes, seldom, and never. Responses were dichotomized into good (always, often, sometimes) and not good (seldom and never) when used as a covariate. We also used questions about health behaviours (i.e. physical activity, diet, alcohol consumption, and tobacco consumption) as covariates since they are linked to both health outcomes^47^ and digital media use^48,49^. All health behaviour covariates were dichotomized when added to the models. Participants were considered physically active if they reported moderate to vigorous activity for at least 1 h per day, 5 days per week, or more. Reports of regular meals were considered a proxy for healthy dietary habits. In the analyses, participants who reported eating breakfast and lunch daily were categorized as having regular meals. Daily tobacco use was defined as reports of daily use of cigarettes or snuff. Heavy alcohol consumption was defined as drinking alcohol every week.

### Statistical analyses

Descriptive statistics of study population sociodemographics and health behaviours (used as covariates), and reported time spent on digital media are expressed as proportions, means and frequencies. To analyse the mean differences, pairwise t-tests with Bonferroni adjustment were performed. Differences within the study population and attrition group were assessed using the Chi-squared test. All statistical tests were conducted at a 5% significance level.

Three different regression models were fitted to calculate odds ratios for each examined health outcome. Univariate binomial logistic regression was used to calculate unadjusted odds ratios, and two multivariate binomial logistic regressions with multiple covariates, Models A and B, were used to calculate adjusted odds ratios. Both Models A and B were adjusted for sociodemographic covariates (gender, high school program, origin, and family finances). Model B was additionally adjusted for health behaviour covariates (physical activity, diet, and alcohol and tobacco consumption). The odds ratios are presented with 95% confidence intervals, where the category “1 h or less” spent on digital media was used as a reference category. Too few adolescents reported using digital media “not at all” to make this time category appropriate as reference category.

To examine gender interactions, we fitted logistic regression models to investigate possible interactions with gender (results not shown). In these analyses, we added an interaction term (composed of gender and reported time spent on different digital media) to Models A and B. We found no evidence of any interactions with gender, and consequently, we present the results for all genders together. All analyses were performed in IBM SPSS Statistics 27.

## Results

Table 1 shows the sociodemographic and health behaviour data. Equal proportions of boys and girls participated in the study (49.2% were boys, 49.8% were girls, and 1% reported other genders). All participants answered the survey during their second year in high school, i.e., participants were generally between 16 and 17 years of age. Most participants were Swedish in origin (76%, which is similar to the Swedish population^50^) and reported strong family finances (78.9%). Less than half of the participants (41.9%) reported being physically active, 29.8% ate regular meals, 13.3% used tobacco daily, and 6.3% consumed alcohol every week. There were 573 individuals with missing data on digital media usage, totally or partially, who have been excluded from the analyses. Compared to those who were included, there were significantly higher proportions of individuals who were boys (56.1 vs. 49.2%, *p* < 0.001), had foreign background (51.0% vs. 24.0%, *p* < 0.001) or reported poor family finances (32.8% vs. 21.1%, *p* < 0.001). Also, there were more adolescents who went to introductory programs (introduction programs in Sweden support students not eligible for national high school programs, aiming to prepare them to either qualify for a national programme or prepare for employment) among those who were excluded (27.5% vs. 0.8%, *p* < 0.001). Generally, there were no differences in digital media usage between those excluded and those who were not. For social media, watching movies/series/video clips, and digital schoolwork there were no differences in usage between the excluded and included. However, for gaming we found a higher proportion who reported “not at all” (45.8% vs. 63.2%, *p* < 0.001) among the excluded compared to the included.

**Table 1:** Sociodemographic and health behaviour data of the study population.

Sociodemographics		% (*n*)			n
Gender	Girl	49.8% (1769)			
	Boy	49.2% (1749)			
	Other gender	1.0% (35)			
	Missing				13
High school program	College preparatory program	61.5% (2185)			
	Vocational programs	30.0% (1065)			
	Introductory program	0.8% (27)			
	Others	7.7% (275)			
	Missing				14
Origin	Swedish	76.0% (2701)			
	Non-Swedish	24.0% (852)			
	Missing				13
Family finance	Poor	21.1% (747)			
	Good	78.9% (2787)			
	Missing				32
Health behaviours		% (n)			
Physically active		41.9% (1483)			
Regular meals		39.8% (1417)			
Daily tobacco user		13.3% (473)			
Alcohol consumption every week		6.3% (224)			

### Time spent on digital media

Table 2 shows the average reported time study participants spent on different digital media platforms. On average, participants reported high daily usage of all digital media categories: social media, movies/series/video clips, schoolwork, and gaming. However, the distribution of time spent across various media platforms differed by gender. Boys reported spending more time on gaming (boys: 2.37 h, SD 2.00; girls: 0.77 h, SD 1.42), while girls reported spending more time on social media (girls: 3.24 h, SD 1.85; boys: 2.62 h, SD 1.89) and slightly more time on schoolwork (girls: 2.85 h, SD 2.06; boys: 2.31 h, (SD 1.67). Participants of other genders reported high usage of all categories of digital media (2.43–3.17 h, SDs: 2.07–2.39).

**Table 2:** Average time per day spent on different digital media.

Means in hours (SD)						
	Girls (a)		Boys (b)		Other (c)	
Social media	3.24 (1.85)	b*	2.62 (1.89)		3.17 (2.39)	
Movies, series, video clips, etc.	2.67 (1.73)	b*	2.50 (1.67)		3.66 (2.30)	a* b*
School work	2.85 (2.06)	b*	2.31 (1.67)		2.50 (2.07)	
Gaming	0.77 (1.42)		2.37 (2.00)	a*	2.43 (2.36)	a*

*Significantly different (*p* < 0.05) from the corresponding value in another gender group. The letters correspond to a specific gender group (a = girls, b = boys, c = other).

Table 3 shows the distribution of reported time spent on the respective digital media categories. Most participants were categorized as moderate, high, or very high users of social media (74.1%), watching movies/series/video clips (71.1%) and digital tools for schoolwork 60.8%). The reported time spent on gaming exhibited a slightly different distribution, as 63.2% were categorized as low users and 32.8% of participants reported not playing digital games at all. Only a few participants reported not using social media or watching movies/series/video clips at all (2.4% and 2.5%, respectively). Almost 3 out of 4 participants (73%) reported spending three hours or more daily with at least one type of digital media (social media, movies/series/video clips, or gaming) in their leisure time. One in ten adolescents in the study (10.1%) reported using each category of digital media 3 h or more per day in their leisure time, and 1.4% reported using each media 6 h or more per day in their leisure time, indicating that adolescents use different digital media simultaneously. The reported time spent on digital tools for schoolwork was high for some participants and low for others (29.0% spent 4–5 h per day or more on digital tools for schoolwork, and 39.2% did not use digital tools for schoolwork at all or less than 1 h).

**Table 3:** Distribution of time spent on different digital media platforms, per time category, per day.

	Low: Not at all/ 1 h. or less, % (*n*)		Moderate: 2–3 h, % (*n*)		High: 4–5 h, % (*n*)		Very high: ≥ 6 h, % (*n*)	
Social media	25.9% (924)		40.4% (1441)		22.0% (784)		11.7% (417)	
Movies, series, video clips etc.	28.9% (1030)		47.2% (1684)		16.0% (572)		7.9% (280)	
Digital schoolwork	39.2% (1397)		31.8% (1135)		16.3% (583)		12.6% (451)	
Gaming	63.2% (2254)		20.2% (719)		10.6% (378)		6.0% (215)	

### Social media and health outcomes

**Fig. 1:**
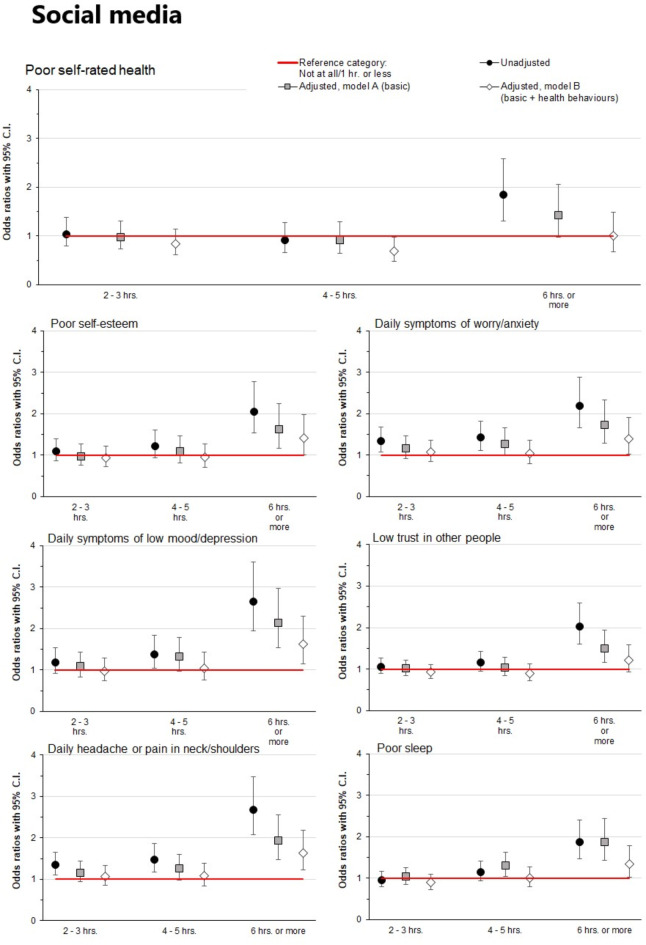
Associations of daily reported time spent on social media with various health outcomes. Models A and B were adjusted for gender, high school program, origin, and family finances. Model B was additionally adjusted for physical activity, regular meals, and alcohol and tobacco consumption. The reference category (0–1 h) is represented in the figure by the red reference line. Associations were considered statistically significant if the 95% confidence intervals did not overlap 1 (the reference category).

Associations of reported time spent on social media with self-rated general and mental health outcomes are shown in Fig. 1 (detailed information is provided in the online Supplementary Table 1). In the unadjusted models, very high levels of reported time spent on social media (≥ 6 h per day) were associated with all investigated adverse health outcomes (poor self-rated health, poor self-esteem, daily symptoms of worry/anxiety and low mood/depression, low trust in other people, poor sleep and daily headache or neck/shoulder pain (ORs 1.84–2.68, CIs 1.31–3.61, all *p* < 0.05) as compared to those who spent one hour or less on social media. High (4–5 h per day) levels of reported time spent on social media were significantly associated with daily symptoms of worry/anxiety, daily symptoms of low/mood/depression, and daily headache or neck/shoulder pain (ORs 1.38–1.48, CIs 1.03–1.87, all *p* < 0.05), while moderate levels (2–3 h per day) were not or only weakly associated with the investigated health outcomes.

All associations were attenuated when adjusted for sociodemographic variables (Model A), and additional adjustment for health behaviours (Model B) attenuated the associations further. Although associations were considerably weaker after adjustments for both sociodemographic variables and reported health behaviours, associations between very high levels of time spent on social media (≥ 6 h per day) and poor self-esteem, daily symptoms of worry/anxiety, daily symptoms of low mood/depression, headache or neck/shoulder pain and poor sleep were still statistically significant (ORs 1.35–1.64, CIs 1.02–2.30, all *p* < 0.05).

### Watching movies, series, video clips, etc., and health outcomes

**Fig. 2:**
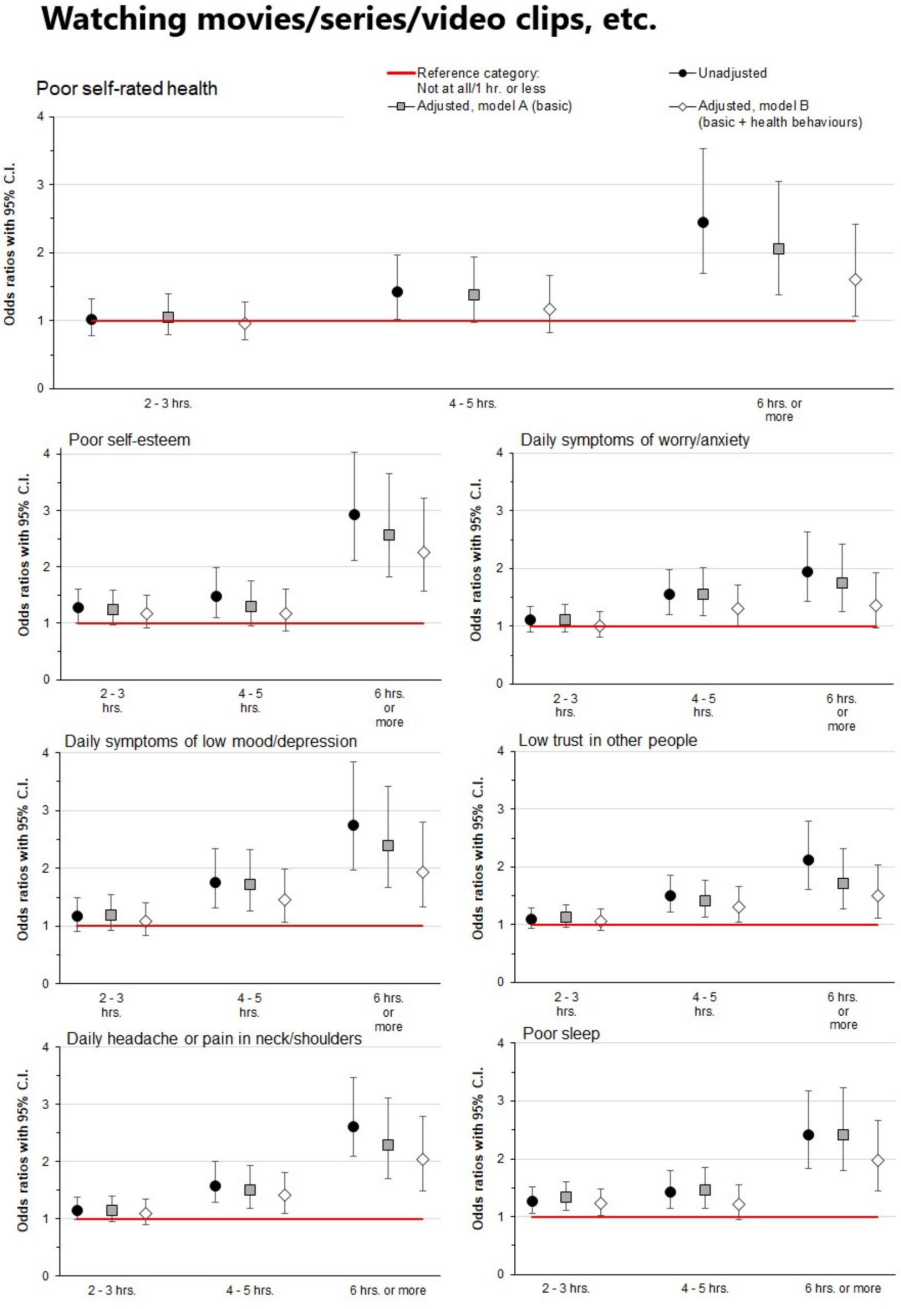
Associations of daily reported time spent watching movies, series, and video clips with various health outcomes. Models A and B were adjusted for gender, high school program, origin, and family finances. Model B was additionally adjusted for physical activity, regular meals, and alcohol and tobacco consumption. The reference category (0–1 h) is represented in the figure by the red reference line. Associations were considered statistically significant if the 95% confidence intervals did not overlap 1 (the reference category).

Figure 2 shows associations of reported time spent watching movies/series/video clips etc., with investigated health outcomes (detailed information is provided in the online Supplementary Table 2). In the unadjusted models, high and very high levels (4–5 h and ≥ 6 h per day) were significantly associated with all adverse health outcomes (ORs 1.42–2.92, CIs 1.02–4.03, all *p* < 0.05), as compared to those who spent one hour or watching movies/series/video clips etc. Moderate levels of time spent on movies, series, video clips, etc. (2–3 h per day) were not associated with adverse health outcomes except poor sleep (OR 1.26, CI 1.06–1.51, *p* = 0.01).

As for social media, the associations were attenuated when adjusted for sociodemographic variables (Model A), and additional adjustment for health behaviours (Model B) attenuated the associations further. However, after adjustments for both sociodemographic variables and health behaviours, spending 6 h or more per day watching movies/series/video clips etc., was still associated with all adverse general and mental health outcomes, except daily worry/anxiety (ORs 1.50–2.25, CIs 1.06–3.22, all *p* < 0.05). Also, high levels (4–5 h) of time were still associated with daily symptoms of low mood/depression, low trust in other people, and headache or neck/shoulder pain after adjustments for sociodemographic variables and health behaviours (ORs 1.46, 1.32 and 1.41, CIs 1.06–1.99, 1.05–1.65 and 1.09–1.82, respectively, all *p* < 0.05).

### Digital tools for schoolwork and health outcomes

**Fig. 3:**
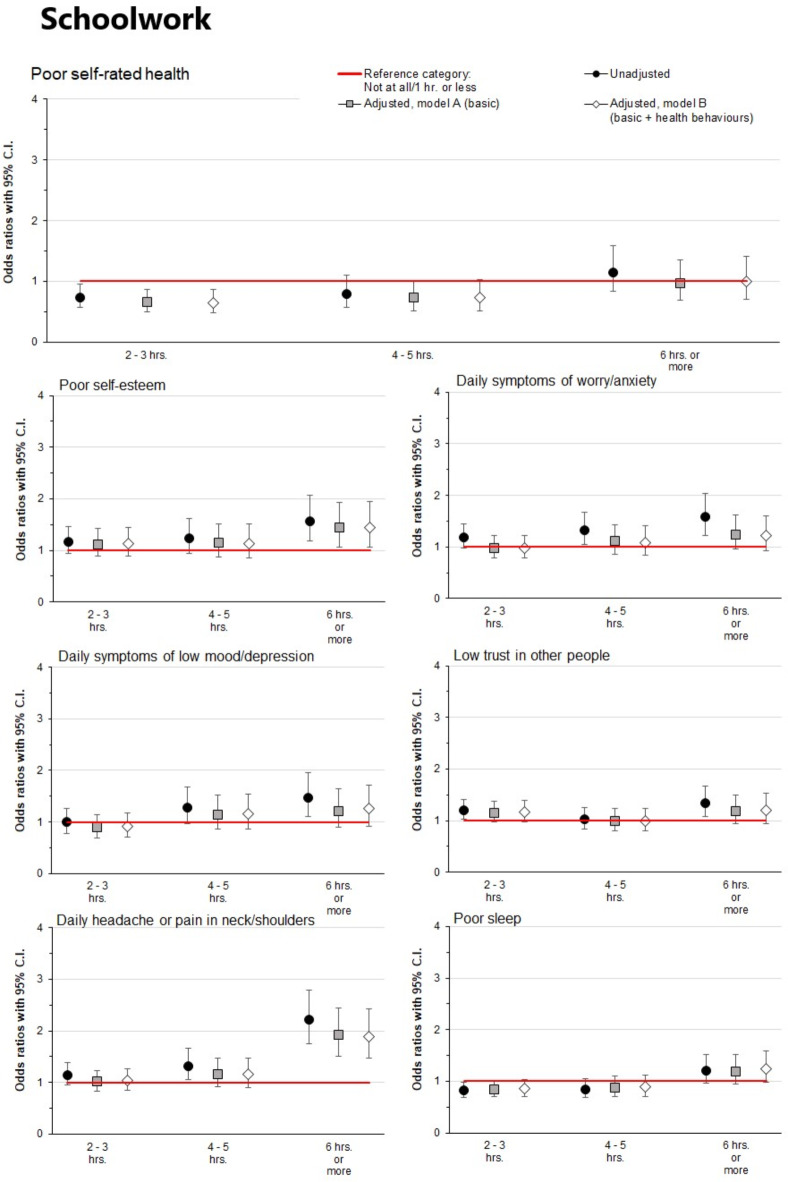
Associations of daily reported time spent on digital tools for schoolwork with various health outcomes. Models A and B were adjusted for gender, high school program, origin, and family finances. Model B was additionally adjusted for physical activity, regular meals, and alcohol and tobacco consumption. The reference category (0–1 h) is represented in the figure by the red reference line. Associations were considered statistically significant if the 95% confidence intervals did not overlap 1 (the reference category).

Figure 3 shows associations between reported time spent on digital tools for schoolwork and the various investigated health outcomes (detailed information is provided in the online Supplementary Table 3). In the unadjusted models, moderate (2–3 h per day) and high (4–5 h per day) levels of reported time spent on digital tools for schoolwork were not associated with any adverse health outcomes compared to those who spent one hour or less. Very high levels (≥ 6 h per day) spent on digital tools for schoolwork were also not, or only weakly, associated with adverse health outcomes, except for daily headache or pain in the neck/shoulders where an association was found (OR 2.22, CI 1.79–2.79, *p* < 0.001). An association was also observed for poor self-esteem (OR 1.57, CI 1.19–2.07, *p* < 0.01). These associations were only weakly attenuated by adjusting for sociodemographic variables (Model A) or sociodemographics and health behaviours (Model B) (ORs 1.90 and 1.44, CIs 1.48–2.43 and 1.06–1.95, respectively).

### Gaming and health outcomes

**Fig. 4:**
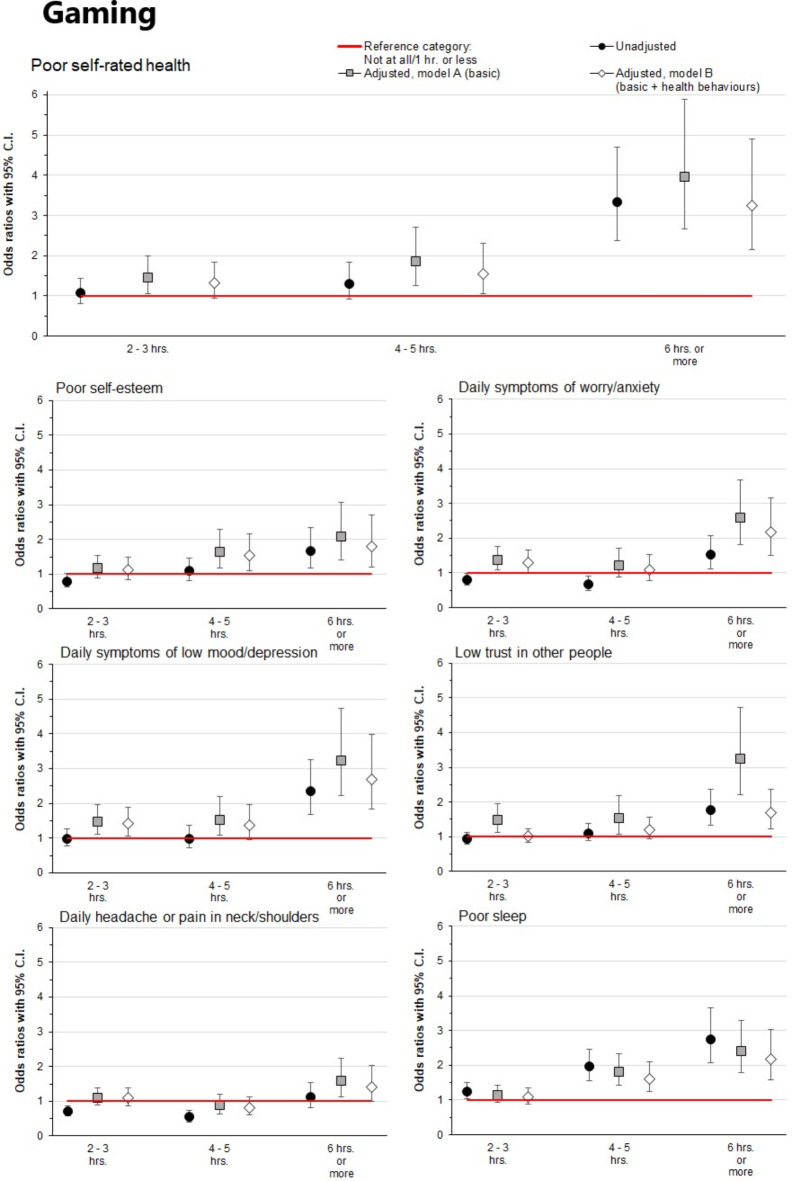
Associations of daily reported time spent on gaming with various health outcomes. Models A and B were adjusted for gender, high school program, origin, and family finances. Model B was additionally adjusted for physical activity, regular meals, and alcohol and tobacco consumption. The reference category (0–1 h) is represented in the figure by the red reference line. Associations were considered statistically significant if the 95% confidence intervals did not overlap 1 (the reference category).

As shown in Fig. 4, we also investigated associations of reported time spent on gaming with various health outcomes (detailed information is provided in the online Supplementary Table 4). In the unadjusted models, very high levels of reported time spent on gaming (≥ 6 h per day) were associated with all adverse health outcomes except daily headache or pain in the neck/shoulders (ORs 1.53–3.34, CIs 1.12–4.70, all *p* < 0.01), as compared to those who spent 1 h or less on gaming. These associations were particularly strong for poor self-rated health and poor sleep (ORs 3.34 and 2.74, CIs 2.06–3.65 and 2.37–4.71, respectively). Moderate (2–3 h per day) and high (4–5 h per day) levels of reported time spent on gaming were not or very weakly associated with adverse health outcomes except for poor sleep (ORs 1.26 and 1.96, CIs 1.04–1.51 and 1.56–2.46, respectively). In contrast to the above-reported associations between time spent on digital media and various health outcomes, the associations between time spent on gaming and health outcomes were relatively unaffected by adjustment for sociodemographic variables (Model A) and sociodemographics and health behaviours (Model B).

## Discussion

In this population-based study, very high levels of time (≥ 6 h per day) spent on digital media (social media, watching movies/series/video clips, or gaming) were associated with most investigated adverse health outcomes (poor self-rated health, poor self-esteem, daily symptoms of worry/anxiety, daily symptoms of low mood/depression, low trust in other people, poor sleep and daily headache or neck/shoulder pain) in the unadjusted models. After adjusting for sociodemographic variables and health behaviours, the strength of the associations weakened considerably (except for gaming), although most associations remained statistically significant. Watching movies/series/video clips 4–5 h or more per day was still associated with adverse outcomes after adjustment for sociodemographic variables and health behaviours. Notably, digital tools for schoolwork showed minimal negative associations with adverse health outcomes, in both unadjusted and adjusted models, except for headache or pain in the neck/shoulders after spending 6 h or more per day.

We found that 73% of participants reported spending three hours or more on at least one type of digital media in their leisure time in 2019. Approximately one in ten of the participants reported using each digital media (social media, movies/series/video clips, and gaming) for three hours or more per day, and 1.4% used each media type 6 h or more per day, which implies that several types of digital media are used simultaneously. These reports on digital media usage are in line with previous reports on Swedish^6^ and US adolescents^51,52^.

Social media was the most widely used of the different types of digital media investigated in this study. Approximately one third of the participants reported using social media more than three hours per day, and approximately 12% reported using social media six hours or more per day. Almost half of the participants reported watching movies/series/video clips etc. between two and three hours daily. Reports of time spent on gaming exhibited the greatest variation across participants, with one third reporting not gaming at all, 17% reported spending more than three hours per day, and 6% reported 6 h or more per day. We also observed gender differences regarding reported time spent on different types of digital media. Boys reported spending more time gaming than girls, and girls spent more time on social media than boys. These gender differences align with previous reports on Swedish adolescents^6^. Reports from US^7^ and Dutch^53^ adolescents also show that girls use social media more frequently than boys do.

We found no or weak associations between reports of spending moderate amounts of time (2–3 h) on digital media (social media, watching movies/series/video clips etc., schoolwork or gaming) and the adverse health outcomes investigated in this study. Likewise, we found no associations between high levels of time spent on digital schoolwork and adverse health outcomes, except for very high levels (≥ 6 h per day), where an association was found with headache or pain in the neck/shoulders (OR:2.22, *p* < 0.001). A comparison with previous literature shows that other studies also have found that extensive use of digital media may cause musculoskeletal symptoms^54–56^. However, the association between digital schoolwork and pain in the neck/shoulders needs to be investigated further in future studies.

Consistent and significant associations with adverse health outcomes for social media and watching movies/series/video clips, respectively, started to appear in the group of participants reporting spending 4–5 h per day and followed a dose-response relationship where the ORs for adverse outcomes increased further in the group spending six hours or more on either of these types of digital media. These results are comparable to the results of a meta-analysis by Zou et al.^20^ that show no associations between depression and watching TV up to 4.5 h a day compared to 1 h a day. The study by Zou et al. also showed that teenagers who play computer games 2 h or more per day have a higher risk of depression compared to those who play less. Several other studies have also shown a dose-response relationship between time spent on digital media in general and symptoms of adverse mental health outcomes, including depression, however, starting at estimates of around 1 h per day^27–29,57,58^. The dose-response association between time spent on digital media and health outcomes highlights the need for public health recommendations and interventions focused on healthy and balanced use of digital media among adolescents.

In an article by Khan et al.^57,^the detrimental relationships between screen time and mental wellbeing were attenuated for those with high physical activity. This is in agreement with our findings that the associations were weaker after adjusting for health behaviours for some digital media categories. Associations of time spent on schoolwork and gaming with health outcomes were not attenuated by adjusting for sociodemographic variables and health behaviours, such as physical activity, diet and alcohol and tobacco consumption. However, the associations were clearly attenuated for social media and watching movies/series/video clips etc. One possible interpretation of the results is that a healthy lifestyle and socioeconomic prosperity may mitigate the adverse effects of screen time on health. Interestingly, previous studies have suggested that high socioeconomic status and healthy behaviours may have a moderating effect on the relationship between screen time and mental ill-health^11,57,59^. This indicates that public health interventions aimed at combating socioeconomic inequities and unhealthy behaviours could, at least in part, mitigate adverse effects of high usage of social media and watching movies/series/video clips etc. Though, in our study, almost all associations between very high levels of time (≥ 6 h per day) spent on social media or watching movies/series/video clips, and adverse health outcomes were statistically significant after adjustment for sociodemographics and health behaviours. This might imply that the adverse associations of very high levels of time (≥ 6 h per day) spent on these types of digital media cannot be mitigated by healthy behaviours or socioeconomic prosperity.

The manner of use of digital media may be more important than the time exposure. To be precise, what adolescents are doing/watching/playing on their digital media devices, how they do it, and how it affects their relationships may matter more than how much time they spend doing it^60,61^. It is well known that digital media can impact health and well-being in both positive and negative directions^8,9^. In our study, digital schoolwork was not associated with adverse health outcomes, except for neck/shoulder pain. This might imply that it is not the screen time itself, and rather the content adolescents are exposed to when they are gaming, using social media or watching video clips that contributes to symptoms of mental ill-health. Associations between time spent on digital media and adverse health outcomes were particularly strong for watching movies/series/video clips in our study. Previous studies have suggested that mentally passive digital media usage might be more related to mental problems than active and interactive usage^11,62^. Therefore, one possible explanation for our findings could be that watching movies/series/video clips, etc., is a more passive activity than gaming and social media usage, and therefore poses a greater risk for health problems. However, the adverse effects of digital media “stealing” time from other activities, such as sleep, physical activity, and school work (so-called displacement effects^24^), remain even if the time spent is perceived as positive. Notably, a review by Sanders et al.. showed that social media use was associated with depression in children and adolescents and had no positive effects, irrespective of content^26^. Nonetheless, further research should address how dose-response associations between time spent on digital media and health might differ depending on content within and usage of the respective digital media.

### Limitations and strengths

As this was a cross-sectional study, the associations between time spent on digital media and adverse health outcomes were correlational. Therefore, we cannot determine whether high usage of digital media leads to health problems or whether people with health problems are inclined to spend more time using digital media. Previous research shows an ongoing “chicken or egg” debate about which came first: high digital media usage, poor mental health, or a combination of both^11^. Previous studies have suggested a joint or bidirectional relationship between screen time and adverse health outcomes^63^. However, there is a growing body of evidence showing that high usage of digital media may “come first”, causing mental health problems^11,64^. Another limitation was the use of self-reported data, which may be subject to recall bias, for example, the individual might not be aware of how much time they spend on digital media. However, considering that this study is based on data from an anonymous survey within the school setting, the use of self-reported data was inevitable. In this survey, adolescents took part in creating the questions to ensure that they were relevant and understandable. Nevertheless, the question about self-rated health in the survey has been proven valid for measuring “general health” adequately^21,43^. Also questions about mental health are similar to the questions in the Kessler-6 screening tool, which provides an accurate and valid measure of mental distress^44,45^ and to the questions in the Health Behaviour in School-aged Children (HBSC) study, a comprehensive international research project on child health and well-being across 51 countries^46^. The survey includes questions about the types of digital media that adolescents primarily use, i.e. social media, gaming, watching movies/series/video clips, and digital schoolwork. However, one limitation of the study is that the survey does not capture other forms of digital media usage, such as online shopping, or web browsing for non-school-related information. This presents an important area for further research. Also, the survey did not capture variations in usage over weekdays and weekends. Instead, the adolescents report an average of time spent during a typical day, which might have influenced the answers. Finally, a few individuals (1.4%) report 24 h or more of total digital media usage per day, which implies multitasking/ use of different digital media simultaneously. It remains unclear whether multitasking could introduce bias in the associations between digital media use and health outcomes, making this an important area for future research.

This study also has several significant strengths. First, it was population-based and contained a relatively large sample size (3566 adolescents). Furthermore, the survey response rate was relatively high (75% of respondents), which increases generalizability. Indeed, it is estimated that 24% of children in the Swedish population have a foreign background^50^, which is identical to what we found in this study (24%). Additionally, since the survey contained a wide range of questions about health and lifestyle, it was possible to comprehensively evaluate associations of digital media with self-rated general and mental health.

## Conclusions

This study adds to the growing evidence showing a dose-response relationship between time spent on digital media and adverse health outcomes. Associations between very high levels of time (≥ 6 h per day) spent on social media, gaming and watching movies/series/video clips were associated with almost all investigated adverse health outcomes in both unadjusted models and models adjusted for sociodemographics and health behaviours. However, associations differed between different digital media. For digital schoolwork there were no significant associations with health outcomes except for very high levels (≥ 6 h per day), where an association was found with headache or pain in the neck/shoulders. Associations with health outcomes were particularly strong for watching movies/series/video clips, etc. and especially with symptoms of low mood/depression and daily headache or neck/shoulder pain, where adverse associations started to appear after 4–5 h. We believe this study can have a meaningful societal impact by providing new insights into how different digital media affect health and offering valuable guidance on usage thresholds linked to adverse effects. Future research should explore whether the reduction of specific types of digital media improves general and mental health in this age group. In addition, public health interventions focused on healthy and balanced use of digital media are warranted.

## Electronic supplementary material

Below is the link to the electronic supplementary material.

Supplementary Material 1

## Data Availability

The data underlying this article are not publicly available due to legal reasons and to protect individuals’ personal information. However, it is possible to contact the corresponding author to obtain information regarding procedures for accessing the data.

## Abbreviations

OR: Odds ratio
CI: Confidence interval

## References

[1] Thapar, A., Eyre, O., Patel, V. & Brent, D. Depression in young people. *Lancet* **400** (10352), 617–631 (2022). 35940184 10.1016/S0140-6736(22)01012-1

[2] The Public Health Agency of Sweden. Health Behaviour in Shoool-aged Children (HBS) Survey, Sweden (2023).

[3] Bertha, E. A. & Balazs, J. Subthreshold depression in adolescence: a systematic review. *Eur. Child. Adolesc. Psychiatry*. **22** (10), 589–603 (2013). 23579389 10.1007/s00787-013-0411-0

[4] The Public Health Agency of Sweden. Research compilation on digital media use and mental, physical and sexual health and lifestyle among children and adolescents (2024).

[5] Mytton, O. T. et al. Changing patterns of health risk in adolescence: implications for health policy. *Lancet Public. Health*. **9** (8), 629–634 (2024). 10.1016/S2468-2667(24)00125-7 38996502

[6] Swedish Agency for the Media. Ungar & medier 2021 (2021).

[7] Pew Research Center. Teens, Social Media and Technology 2022 (2022).

[8] Allcott, H., Braghieri, L., Eichmeyer, S. & Gentzkow, M. The welfare effects of social media. *Am. Econ. Rev.* **110**, 3 (2020).

[9] Reid Chassiakos, Y. L., Radesky, J., Christakis, D., Moreno, M. A. & Cross, C. Council on C, Media. Children and adolescents and digital media. *Pediatrics*. **138**(5). (2016). 10.1542/peds.2016-2593 27940795

[10] Dibben, G. O. et al. Adolescents’ interactive electronic device use, sleep and mental health: a systematic review of prospective studies. *J. Sleep. Res.* **32** (5), 138–199 (2023). 10.1111/jsr.13899 PMC10909457 37029099

[11] Tang, S., Werner-Seidler, A., Torok, M., Mackinnon, A. J. & Christensen, H. The relationship between screen time and mental health in young people: a systematic review of longitudinal studies. *Clin. Psychol. Rev.* **86**, 102–121 (2021). 10.1016/j.cpr.2021.102021 33798997

[12] Zhang, J. et al. An updated of meta-analysis on the relationship between mobile phone addiction and sleep disorder. *J. Affect. Disord*. **305**, 94–101 (2022). 35149136 10.1016/j.jad.2022.02.008

[13] de Valle, M. K., Gallego-Garcia, M., Williamson, P. & Wade, T. D. Social media, body image, and the question of causation: meta-analyses of experimental and longitudinal evidence. *Body Image*. **39**, 276–292 (2021). 34695681 10.1016/j.bodyim.2021.10.001

[14] Jeronimo, F. & Carraca, E. V. Effects of fitspiration content on body image: a systematic review. *Eat. Weight Disord.*. **27** (8), 3017–3035 (2022). 36401082 10.1007/s40519-022-01505-4 PMC9676749

[15] Girela-Serrano, B. M. et al. Impact of mobile phones and wireless devices use on children and adolescents’ mental health: a systematic review. *Eur. Child. Adolesc. Psychiatry*. **33** (6), 1621–1651 (2024). 35705765 10.1007/s00787-022-02012-8 PMC9200624

[16] Poorolajal, J., Sahraei, F., Mohamdadi, Y., Doosti-Irani, A. & Moradi, L. Behavioral factors influencing childhood obesity: a systematic review and meta-analysis. *Obes. Res. Clin. Pract.* **14** (2), 109–118 (2020). 32199860 10.1016/j.orcp.2020.03.002

[17] Shin, M., Juventin, M., Wai Chu, J., Manor, Y. & Kemps, E. Online media consumption and depression in young people: a systematic review and meta-analysis. *Comput. Hum. Behav.* **128**, 14 (2022).

[18] Tholl, C., Bickmann, P., Wechsler, K., Frobose, I. & Grieben, C. Musculoskeletal disorders in video gamers—a systematic review. *BMC Musculoskelet. Disord*. **23** (1), 678 (2022). 35842605 10.1186/s12891-022-05614-0 PMC9288077

[19] Yue, C. et al. Dose-response relationship between daily screen time and the risk of low back pain among children and adolescents: a meta-analysis of 57831 participants. *Environ. Health Prev. Med.* **28**, 64 (2023). 37899211 10.1265/ehpm.23-00177 PMC10613558

[20] Zou, Z., Xiang, J., Wang, H., Wen, Q. & Luo, X. Association of screen time-based sedentary behavior and the risk of depression in children and adolescents: dose-response meta-analysis. *Arch. Clin. Psychiatry*. **48** (6), 235–244 (2021).

[21] DeSalvo, K. B., Bloser, N., Reynolds, K., He, J. & Muntner, P. Mortality prediction with a single general self-rated health question. A meta-analysis. *J. Gen. Intern. Med.* **21** (3), 267–275 (2006). 16336622 10.1111/j.1525-1497.2005.00291.x PMC1828094

[22] Thomas, K. et al. Associations of Psychosocial factors with multiple Health behaviors: a Population-based study of Middle-aged men and women. *Int. J. Environ. Res. Public. Health*. **17**(4). (2020). 10.3390/ijerph17041239 PMC7068361 32075162

[23] Marmot, M. & Wilkinson, G. *Social Determinants of Health* (Oxford University Press, 2000).

[24] Neuman, S. B. The displacement effect: assessing the relation between television viewing and reading performance. *Read. Res. Q.* **23** (4), 414–440 (1988).

[25] Santos, R. M. S. et al. The associations between screen time and mental health in adolescents: a systematic review. *BMC Psychol.* **11** (1), 127 (2023). 37081557 10.1186/s40359-023-01166-7 PMC10117262

[26] Sanders, T. et al. An umbrella review of the benefits and risks associated with youths’ interactions with electronic screens. *Nat. Hum. Behav.* **8** (1), 82–99 (2024). 37957284 10.1038/s41562-023-01712-8

[27] Kelly, Y., Zilanawala, A., Booker, C. & Sacker, A. Social media use and adolescent mental health: findings from the UK Millennium Cohort Study. *EClinicalMedicine* **6**, 59–68 (2018). 31193561 10.1016/j.eclinm.2018.12.005 PMC6537508

[28] Liu, M., Wu, L. & Yao, S. Dose-response association of screen time-based sedentary behaviour in children and adolescents and depression: a meta-analysis of observational studies. *Br. J. Sports Med.* **50** (20), 1252–1258 (2016). 26552416 10.1136/bjsports-2015-095084 PMC4977203

[29] Eirich, R. et al. Association of screen time with internalizing and externalizing behavior problems in children 12 years or younger: a systematic review and meta-analysis. *JAMA Psychiatry*. **79** (5), 393–405 (2022). 35293954 10.1001/jamapsychiatry.2022.0155 PMC8928099

[30] Ferguson, C. J. Everything in moderation: moderate use of Screens unassociated with child behavior problems. *Psychiatr Q.* **88** (4), 797–805 (2017). 28168645 10.1007/s11126-016-9486-3

[31] Hrafnkelsdottir, S. M. et al. Less screen time and more frequent vigorous physical activity is associated with lower risk of reporting negative mental health symptoms among Icelandic adolescents. *PLoS One*. **13** (4), e0196286 (2018). 29698499 10.1371/journal.pone.0196286 PMC5919516

[32] Keyes, K. M., Gary, D., O’Malley, P. M., Hamilton, A. & Schulenberg, J. Recent increases in depressive symptoms among US adolescents: trends from 1991 to 2018. *Soc. Psychiatry Psychiatr. Epidemiol.* **54** (8), 987–996 (2019). 30929042 10.1007/s00127-019-01697-8 PMC7015269

[33] Orben, A. & Przybylski, A. K. The association between adolescent well-being and digital technology use. *Nat. Hum. Behav.* **3** (2), 173–182 (2019). 30944443 10.1038/s41562-018-0506-1

[34] Riehm, K. E. et al. Associations between time spent using social media and internalizing and externalizing problems among US Youth. *JAMA Psychiatry*. **76** (12), 1266–1273 (2019). 31509167 10.1001/jamapsychiatry.2019.2325 PMC6739732

[35] MacKenzie, M. D., Scott, H., Reid, K. & Gardani, M. Adolescent perspectives of bedtime social media use: a qualitative systematic review and thematic synthesis. *Sleep. Med. Rev.* **63**, 101626 (2022). 35468519 10.1016/j.smrv.2022.101626

[36] Region Östergötland. The youth survey Om mig (About me). https://www.regionostergotland.se/ommig (2024).

[37] Hale, L. & Guan, S. Screen time and sleep among school-aged children and adolescents: a systematic literature review. *Sleep. Med. Rev.* **21**, 50–58 (2015). 25193149 10.1016/j.smrv.2014.07.007 PMC4437561

[38] Perez, O. et al. Validated assessment tools for screen media use: a systematic review. *PLoS One*. **18** (4), e0283714 (2023). 37053175 10.1371/journal.pone.0283714 PMC10101444

[39] Chau, K., Bhattacherjee, A., Senapati, A., Guillemin, F. & Chau, N. Association between screen time and cumulating school, behavior, and mental health difficulties in early adolescents: a population-based study. *Psychiatry Res.* **310**, 114467 (2022). 35227988 10.1016/j.psychres.2022.114467

[40] Weatherson, K. et al. Complete mental health status and associations with physical activity, screen time, and sleep in youth. *Ment. Health Phys. Act.* **19**, 100354 (2020).

[41] Khan, A. & Burton, N. W. Is physical inactivity associated with depressive symptoms among adolescents with high screen time? Evidence from a developing country. *Ment. Health Phys. Act.* **12**, 94–99 (2017).

[42] Allen, C. D., McNeely, C. A. & Orme, J. G. Self-rated health across race, ethnicity, and immigration status for US adolescents and young adults. *J. Adolesc. Health*. **58** (1), 47–56 (2016). 26552738 10.1016/j.jadohealth.2015.09.006

[43] Chandola, T. & Jenkinson, C. Validating self-rated health in different ethnic groups. *Ethn. Health*. **5** (2), 151–159 (2000). 10984833 10.1080/713667451

[44] Kessler, R. C. et al. Screening for serious mental illness in the general population. *Arch. Gen. Psychiatry*. **60** (2), 184–189 (2003). 12578436 10.1001/archpsyc.60.2.184

[45] Prochaska, J., Sung, H., Max, W., Shi, Y. & Ong, M. Validity study of the K6 scale as a measure of moderate mental distress based on mental health treatment need and utilization. *Int. J. Methods Psychiatr. Res.* **21** (2), 88–97 (2012). 22351472 10.1002/mpr.1349 PMC3370145

[46] Inchley, J., Currie, D., Cosma, A. & Samdal, O. *Health Behaviour in School-aged Children (HBSC) Study Protocol: Background, Methodology and Mandatory Items for the 2017/18 Survey* (CAHRU, 2018).

[47] GBD Risk Factors Collaborators. Global burden of 87 risk factors in 204 countries and territories, 1990–2019: a systematic analysis for the global burden of disease study 2019. *Lancet* **396** (10258), 1223–1249 (2020). 33069327 10.1016/S0140-6736(20)30752-2 PMC7566194

[48] Purba, A. K. et al. Social media use and health risk behaviours in young people: systematic review and meta-analysis. *BMJ* **383**, e073552 (2023). 38030217 10.1136/bmj-2022-073552 PMC10685288

[49] Luu, N. M., Phan, T. H., Oh, J. K. & Myung, S. K. Exposure to electronic cigarette advertisements and use of electronic cigarettes: a meta-analysis of prospective studies. *Nicotine Tob. Res.* **25** (5), 983–990 (2023). 36426864 10.1093/ntr/ntac266

[50] Statistics Sweden. Living conditions for children with a foreign background 26 September 2024. https://www.scb.se/contentassets/ed22f.

[51] Rideout, V. & Robb, M. The Common Sense census: Media use by tweens and teens (2019).

[52] Sanchez, G. & Jenkins, J. H. Social media & subjectivity: adolescent lived experiences with social media in a Southern California middle school. *Soc. Sci. Med.* **348**, 116839 (2024). 38581816 10.1016/j.socscimed.2024.116839

[53] You, Y. Y., Yang-Huang, J., Raat, H. & van Grieken, A. Factors of heavy social media use among 13-year-old adolescents on weekdays and weekends. *World J. Pediatr.* **19** (4), 378–389 (2023). 36806096 10.1007/s12519-023-00690-1 PMC10060361

[54] Warda, D. G., Nwakibu, U. & Nourbakhsh, A. Neck and upper extremity musculoskeletal symptoms secondary to maladaptive postures caused by cell phones and backpacks in school-aged children and adolescents. *Healthc. (Basel)* ;**11**(6). (2023). 10.3390/healthcare11060819 PMC10048647 36981476

[55] Priftis, N. & Panagiotakos, D. Screen time and its Health consequences in children and adolescents. *Child. (Basel)* ;**10**(10). (2023). 10.3390/children10101665 PMC10605067 37892328

[56] Cheung, M. C., Lai, J. S. K., Yip, J. & Cheung, J. P. Y. Increased computer use is Associated with trunk asymmetry that negatively impacts Health-related quality of life in early adolescents. *Patient Prefer. Adher.*. **15**, 2289–2302 (2021). 10.2147/PPA.S329635 PMC8502057 34675493

[57] Khan, A., Lee, E. Y., Rosenbaum, S., Khan, S. R. & Tremblay, M. S. Dose-dependent and joint associations between screen time, physical activity, and mental wellbeing in adolescents: an international observational study. *Lancet Child. Adolesc. Health*. **5** (10), 729–738 (2021). 34384543 10.1016/S2352-4642(21)00200-5

[58] Liu, M. et al. Time spent on social media and risk of depression in adolescents: a dose-response meta-analysis. *Int. J. Environ. Res. Public. Health*. **19**(9). (2022). 10.3390/ijerph19095164 PMC9103874 35564559

[59] Bohnert, M. & Gracia, P. Digital use and socioeconomic inequalities in adolescent well-being: longitudinal evidence on socioemotional and educational outcomes. *J. Adolesc.* **95** (6), 1179–1194 (2023). 37221652 10.1002/jad.12193

[60] Clark, J. L., Algoe, S. B. & Green, M. C. Social network sites and well-being: the role of social connection. *Curr. Dir. Psychol. Sci.* **27** (1), 32–37 (2018).

[61] Waytz, A. & Gray, K. Does online technology make us more or less sociable? A preliminary review and call for research. *Perspect. Psychol. Sci.* **13** (4), 473–491 (2018). 29758166 10.1177/1745691617746509

[62] Hallgren, M., Dunstan, D. W. & Owen, N. Passive versus mentally active sedentary behaviors and depression. *Exerc. Sport Sci. Rev.* **48** (1), 20–27 (2020). 31663866 10.1249/JES.0000000000000211

[63] Zink, J., Belcher, B. R., Imm, K. & Leventhal, A. M. The relationship between screen-based sedentary behaviors and symptoms of depression and anxiety in youth: a systematic review of moderating variables. *BMC Public. Health*. **20** (1), 472 (2020). 32272906 10.1186/s12889-020-08572-1 PMC7147040

[64] Twenge, J. M., Joiner, T. E., Rogers, M. L. & Martin, G. N. Increases in depressive symptoms, suicide-related outcomes, and suicide rates among U.S. adolescents after 2010 and links to increased new media screen time. *Clin. Psychol. Sci.* **6** (1), 3–17 (2018).

