# Supplementary material for: Associations of time spent on different types of digital media with self-rated general and mental health in Swedish adolescents
Source: Sci Rep. 2025 Jan 6;15:993. doi: 10.1038/s41598-024-83951-x (PMC11704018; doi:10.1038/s41598-024-83951-x)
Supplement: Supplementary file 1 — Supplementary Material 1 [file 41598_2024_83951_MOESM1_ESM.pdf]

Supplementary Tables

Associations of time spent on different types of digital media with self rated general and mental health in Swedish adolescents

Helena Frielingsdorf [1], Victoria Fomichov[2], Ingrid Rystedt [1,2] Sofia Lindstrand [1,2], Laura Korhonen [3, 4, 5], Hanna Henriksson [1,2 *]

[1] Department of Health, Medicine and Caring Sciences, Linköping University, Linköping, Sweden

[2] Unit for Strategic Healthcare, Region Östergötland, Linköping, Sweden

[3] Barnafrid and Department of Biomedical and Clinical Sciences, Linköping University, Linköping, Sweden

[4] Center for Social and Affective Neuroscience and Department of Biomedical and Clinical Sciences, Linköping University, Linköping, Sweden

[5] Department of Child and Adolescent Psychiatry and Department of Biomedical and Clinical Sciences, Linköping University, Linköping, Sweden

* Corresponding author: Hanna Henriksson, Unit for Strategic Healthcare, Region Östergötland, 585 81 Linköping, Sweden; Telephone number: +46 703 86 36 38, Email: hanna.henriksson@regionostergotland.se

Supplementary Table 1. Associations of daily reported time spent on social media with various health outcomes, presented as odds ratios (OR) with 95% confidence intervals (CI)										
		OR			95% CI					
**Health outcome**	**Time spent**	**Unadjusted**	**Model A**	**Model B**	**Unadjusted**		**Model A**		**Model B**	
		**OR**	**OR**	**OR**	**LL**	**UL**	**LL**	**UL**	**LL**	**UL**
**Poor self-rated health**	2 - 3 hrs.	1.04	0.97	0.84	0.79	1.38	0.73	1.30	0.62	1.13
	4 - 5 hrs.	0.91	0.92	0.68	0.66	1.27	0.65	1.29	0.48	0.98
	≥ 6 hrs.	1.84	1.42	1.00	1.31	2.59	0.98	2.06	0.68	1.48
**Poor self-esteem**	2 - 3 hrs.	1.10	0.98	0.94	0.86	1.40	0.76	1.27	0.72	1.22
	4 - 5 hrs.	1.23	1.09	0.95	0.93	1.61	0.82	1.46	0.70	1.28
	≥ 6 hrs.	2.06	1.63	1.41	1.53	2.77	1.18	2.26	1.01	1.98
**Daily symptoms of worry/anxiety**	2 - 3 hrs.	1.35	1.16	1.07	1.08	1.67	0.92	1.47	0.85	1.36
	4 - 5 hrs.	1.42	1.27	1.03	1.11	1.82	0.98	1.66	0.79	1.36
	≥ 6 hrs.	2.19	1.73	1.39	1.66	2.88	1.28	2.34	1.02	1.90
**Daily symptoms of low mood/ depression**	2 - 3 hrs.	1.18	1.10	0.98	0.91	1.53	0.84	1.44	0.74	1.30
	4 - 5 hrs.	1.38	1.32	1.05	1.03	1.85	0.98	1.79	0.76	1.44
	≥ 6 hrs.	2.65	2.14	1.63	1.95	3.61	1.53	2.98	1.15	2.30
**Low trust in other people**	2 - 3 hrs.	1.06	1.01	0.92	0.89	1.26	0.84	1.21	0.77	1.12
	4 - 5 hrs.	1.17	1.04	0.90	0.96	1.42	0.84	1.29	0.72	1.12
	6 ≥ hrs.	2.04	1.49	1.21	1.60	2.59	1.15	1.94	0.92	1.58
**Daily headache or neck/ shoulder pain**	2 - 3 hrs.	1.35	1.16	1.07	1.10	1.65	0.94	1.44	0.86	1.34
	4 - 5 hrs.	1.48	1.26	1.08	1.18	1.87	0.98	1.60	0.84	1.39
	6 ≥ hrs.	2.68	1.94	1.64	2.07	3.47	1.47	2.56	1.23	2.18
**Poor sleep**	2 - 3 hrs.	0.96	1.04	0.90	0.80	1.16	0.85	1.26	0.73	1.10
	4 - 5 hrs.	1.15	1.30	1.00	0.93	1.42	1.04	1.63	0.79	1.27
	6 ≥ hrs.	1.88	1.87	1.35	1.48	2.40	1.44	2.44	1.02	1.78
0-1 hours per day was the reference category. Models A and B were adjusted for gender, high school program, origin and family finances. Models B were additionally adjusted for physical activity, regular meals, and alcohol and tobacco consumption. LL: lower limit of the 95% confidence interval. UL: upper limit of the 95% confidence interval.										

Supplementary Table 2. Associations of daily reported time spent on watching TV, movies, video clips etc. with various health outcomes, presented as odds ratios (OR) with 95% confidence intervals (CI)										
		OR			95% CI					
**Health outcome**	**Time spent**	**Unadjusted**	**Model A**	**Model B**	**Unadjusted**		**Model A**		**Model B**	
		**OR**	**OR**	**OR**	**LL**	**UL**	**LL**	**UL**	**LL**	**UL**
**Poor self-rated health**	2 - 3 hrs.	1.01	1.05	0.95	0.77	1.33	0.79	1.39	0.71	1.27
	4 - 5 hrs.	1.42	1.37	1.17	1.02	1.97	0.98	1.94	0.82	1.66
	≥ 6 hrs.	2.44	2.05	1.60	1.69	3.52	1.38	3.04	1.06	2.42
**Poor self-esteem**	2 - 3 hrs.	1.28	1.24	1.18	1.01	1.61	0.98	1.58	0.92	1.50
	4 - 5 hrs.	1.48	1.29	1.17	1.11	1.98	0.95	1.76	0.86	1.60
	≥ 6 hrs.	2.92	2.58	2.25	2.12	4.03	1.82	3.65	1.58	3.22
**Daily symptoms of worry/anxiety**	2 - 3 hrs.	1.10	1.12	1.01	0.90	1.35	0.91	1.38	0.82	1.26
	4 - 5 hrs.	1.55	1.55	1.31	1.21	1.98	1.19	2.01	1.00	1.72
	≥ 6 hrs.	1.95	1.75	1.36	1.44	2.64	1.26	2.43	0.97	1.92
**Daily symptoms of low mood/ depression**	2 - 3 hrs.	1.17	1.20	1.08	0.92	1.50	0.93	1.54	0.83	1.40
	4 - 5 hrs.	1.75	1.71	1.46	1.31	2.34	1.26	2.32	1.06	1.99
	≥ 6 hrs.	2.75	2.39	1.93	1.96	3.85	1.67	3.43	1.33	2.80
**Low trust in other people**	2 - 3 hrs.	1.10	1.13	1.07	0.93	1.29	0.95	1.34	0.90	1.27
	4 - 5 hrs.	1.50	1.42	1.32	1.21	1.85	1.14	1.78	1.05	1.65
	≥ 6 hrs.	2.12	1.72	1.50	1.60	2.79	1.28	2.31	1.11	2.04
**Daily headache or neck/ shoulder pain**	2 - 3 hrs.	1.14	1.15	1.10	0.94	1.38	0.94	1.39	0.90	1.34
	4 - 5 hrs.	1.58	1.51	1.41	1.24	2.00	1.18	1.93	1.09	1.82
	≥ 6 hrs.	2.61	2.30	2.04	1.96	3.46	1.70	3.11	1.49	2.79
**Poor sleep**	2 - 3 hrs.	1.26	1.34	1.22	1.06	1.51	1.11	1.61	1.01	1.48
	4 - 5 hrs.	1.43	1.45	1.21	1.14	1.80	1.15	1.84	0.95	1.55
	≥ 6 hrs.	2.41	2.41	1.97	1.83	3.18	1.80	3.23	1.45	2.67
0-1 hours per day was the reference category. Models A and B were adjusted for gender, high school program, origin and family finances. Models B were additionally adjusted for physical activity, regular meals, and alcohol and tobacco consumption. LL: lower limit of the 95% confidence interval. UL: upper limit of the 95% confidence interval.										

Supplementary Table 3. Associations of daily reported time spent on digital tools for schoolwork with various health outcomes, presented as odds ratios (OR) with 95% confidence intervals (CI)

		**OR**			**95% CI**					
**Health outcome**	**Time spent**	**Unadjusted**	**Model A**	**Model B**	**Unadjusted**		**Model A**		**Model B**	
		**OR**	**OR**	**OR**	**LL**	**UL**	**LL**	**UL**	**LL**	**UL**
**Poor self-rated health**	2 - 3 hrs.	0.74	0.66	0.65	0.57	0.96	0.50	0.87	0.48	0.86
	4 - 5 hrs.	0.80	0.73	0.73	0.58	1.10	0.52	1.02	0.52	1.03
	≥ 6 hrs.	1.15	0.96	0.99	0.83	1.59	0.69	1.35	0.70	1.41
**Poor self-esteem**	2 - 3 hrs.	1.17	1.12	1.13	0.93	1.46	0.89	1.42	0.89	1.44
	4 - 5 hrs.	1.24	1.14	1.13	0.94	1.62	0.86	1.52	0.85	1.50
	≥ 6 hrs.	1.57	1.44	1.44	1.19	2.07	1.07	1.93	1.06	1.95
**Daily symptoms of worry/anxiety**	2 - 3 hrs.	1.19	0.98	0.98	0.97	1.45	0.80	1.21	0.79	1.22
	4 - 5 hrs.	1.32	1.11	1.09	1.04	1.68	0.86	1.43	0.84	1.41
	≥ 6 hrs.	1.58	1.25	1.22	1.23	2.04	0.95	1.63	0.93	1.61
**Daily symptoms of low mood/ depression**	2 - 3 hrs.	0.99	0.89	0.91	0.78	1.26	0.70	1.14	0.70	1.17
	4 - 5 hrs.	1.27	1.14	1.15	0.97	1.67	0.86	1.52	0.86	1.54
	≥ 6 hrs.	1.47	1.22	1.25	1.10	1.96	0.90	1.65	0.91	1.72
**Low trust in other people**	2 - 3 hrs.	1.20	1.15	1.17	1.02	1.42	0.97	1.37	0.98	1.40
	4 - 5 hrs.	1.02	0.99	1.00	0.83	1.25	0.80	1.23	0.80	1.24
	≥ 6 hrs.	1.34	1.18	1.20	1.07	1.66	0.94	1.49	0.95	1.52
**Daily headache or neck/ shoulder pain**	2 - 3 hrs.	1.14	1.02	1.04	0.95	1.38	0.83	1.24	0.85	1.27
	4 - 5 hrs.	1.32	1.16	1.16	1.05	1.66	0.92	1.47	0.91	1.47
	≥ 6 hrs.	2.22	1.92	1.90	1.76	2.79	1.50	2.45	1.48	2.43
**Poor sleep**	2 - 3 hrs.	0.82	0.84	0.86	0.69	0.98	0.70	1.01	0.71	1.04
	4 - 5 hrs.	0.85	0.89	0.89	0.69	1.06	0.71	1.11	0.70	1.12
	≥ 6 hrs.	1.21	1.19	1.25	0.96	1.51	0.94	1.51	0.97	1.59

0-1 hours per day was the reference category. Models A and B were adjusted for gender, high school program, origin and family finances. Models B were additionally adjusted for physical activity, regular meals, and alcohol and tobacco consumption. LL: lower limit of the 95% confidence interval. UL: upper limit of the 95% confidence interval.

Supplementary Table 4. Associations of daily reported time spent on gaming with various health outcomes, presented as odds ratios (OR) with 95% confidence intervals (CI)

		OR			95% CI					
**Health outcome**	**Time spent**	**Unadjusted**	**Model A**	**Model B**	**Unadjusted**		**Model A**		**Model B**	
		**OR**	**OR**	**OR**	**LL**	**UL**	**LL**	**UL**	**LL**	**UL**
**Poor self-rated health**	2 - 3 hrs.	1.07	1.45	1.32	0.80	1.43	1.05	2.00	0.95	1.83
	4 - 5 hrs.	1.29	1.85	1.55	0.91	1.84	1.26	2.72	1.05	2.30
	≥ 6 hrs.	3.34	3.97	3.25	2.37	4.71	2.68	5.88	2.16	4.89
**Poor self-esteem**	2 - 3 hrs.	0.80	1.17	1.11	0.62	1.02	0.88	1.54	0.84	1.48
	4 - 5 hrs.	1.10	1.65	1.54	0.81	1.47	1.18	2.30	1.10	2.16
	≥ 6 hrs.	1.66	2.08	1.80	1.18	2.33	1.40	3.07	1.20	2.70
**Daily symptoms of worry/ anxiety**	2 - 3 hrs.	0.80	1.38	1.29	0.65	1.00	1.08	1.76	1.00	1.66
	4 - 5 hrs.	0.68	1.23	1.10	0.50	0.91	0.88	1.71	0.78	1.54
	≥ 6 hrs.	1.53	2.58	2.17	1.12	2.08	1.80	3.69	1.50	3.15
**Daily symptoms of low mood/ depression**	2 - 3 hrs.	0.98	1.48	1.41	0.77	1.26	1.12	1.95	1.06	1.88
	4 - 5 hrs.	0.99	1.53	1.36	0.72	1.37	1.07	2.19	0.95	1.96
	≥ 6 hrs.	2.34	3.24	2.70	1.68	3.26	2.22	4.72	1.82	3.99
**Low trust in other people**	2 - 3 hrs.	0.95	1.48	1.01	0.80	1.13	1.12	1.95	0.83	1.24
	4 - 5 hrs.	1.11	1.53	1.20	0.88	1.38	1.07	2.19	0.93	1.56
	≥ 6 hrs.	1.77	3.24	1.70	1.32	2.38	2.22	4.72	1.22	2.38
**Daily headache or neck/ shoulder pain**	2 - 3 hrs.	0.71	1.11	1.09	0.58	0.87	0.88	1.39	0.86	1.37
	4 - 5 hrs.	0.54	0.88	0.82	0.41	0.73	0.64	1.20	0.60	1.12
	≥ 6 hrs.	1.12	1.58	1.42	0.82	1.53	1.12	2.23	1.00	2.03
**Poor sleep**	2 - 3 hrs.	1.26	1.15	1.09	1.04	1.51	0.94	1.42	0.88	1.35
	4 - 5 hrs.	1.96	1.81	1.61	1.56	2.46	1.41	2.33	1.24	2.09
	≥ 6 hrs.	2.74	2.42	2.18	2.06	3.65	1.77	3.30	1.58	3.02

0-1 hours per day was the reference category. Models A and B were adjusted for gender, high school program, origin and family finances. Models B were additionally adjusted for physical activity, regular meals, and alcohol and tobacco consumption. LL: lower limit of the 95% confidence interval. UL: upper limit of the 95% confidence interval.
